# Experience of health care at a reference centre as reported by patients and parents of children with rare conditions

**DOI:** 10.1186/s13023-021-01708-5

**Published:** 2021-02-04

**Authors:** Monica Hytiris, Daisy Johnston, Shannon Mullen, Arlene Smyth, Elizabeth Dougan, Martina Rodie, S. Faisal Ahmed

**Affiliations:** 1 Office for Rare Conditions, School of Medicine, Dentistry and Nursing, University of Glasgow, Glasgow, UK; 2 Royal Hospital for Children, University of Glasgow, Glasgow, G51 4TF UK

**Keywords:** Benchmarks, Paediatrics, Patient experience, Rare disease

## Abstract

**Background:**

Whilst diagnostic pathways for children with rare conditions have shown marked improvement, concerns remain about the care children with rare conditions receive at the level of the health care provider. There is, therefore, a need to improve our understanding of the health care received and explore the development of benchmarks that can be regularly monitored.

**Methods:**

Patients and parents with rare conditions at a tertiary children’s hospital were approached to complete a questionnaire-based survey that enquired on their experience of clinical care. The survey explored six key themes: diagnosis; provision of information; availability of support; satisfaction with healthcare team; awareness and support for life-limiting conditions; and participation in research.

**Results:**

130 questionnaires were completed on behalf of 134 patients between 2018 and 2020. Of these, 114 (85%) had received a formal diagnosis, 5 (4%) had a suspected diagnosis and 15 (11%) were undiagnosed. Of the 114 who had received a diagnosis, 24 (20%) were diagnosed within 6 months of developing symptoms, and 22 (20%) within 1–3 years. Seventy patients (53%) reported that they were given little or no information around the time of diagnosis, whilst 81 (63%) felt they were currently well supported, mostly from family members, followed by friends, hospital services, school, other community based healthcare services and lastly, primary care. Of the 127 who were asked, 88 (69%) reported a consistent team of healthcare professionals taking overall responsibility for their care, 86 (67%) felt part of the team, 74 (58%) were satisfied with the level of knowledge of the professionals, and 86 (68%) knew who to contact regarding their condition. Of the 91 who were asked, 23 (25%) were aware their child had a life limiting condition, but only 4 (17%) were receiving specialist support for this. Of 17 who were asked about research, 4 (24%) were actively participating in research, whilst the remainder were all willing to participate in future research.

**Conclusions:**

The survey provides a unique insight into the experience of patients and parents within a specialist centre and the benchmarks that it has revealed can be used for future improvement in services.

## Background

Rare diseases, or conditions, are defined as those that affect less than 1 in 2000 of the population [1]. There are probably around 8000 discrete rare conditions that affect 3 million people in the United Kingdom (UK) [2, 3] and around 75% of these may be children and can have life-limiting or disabling effects [4, 5]. Many diseases are chronic, offer no effective treatment and are frequently incurable [5]. The rare disease plans that have been developed by several countries provide important guidance for improving health and social care but their implementation is uneven across countries [6] and the effect of the implementation has rarely been measured at the level of a service provider.

Although scientific and medical advances have led to marked improvements in reaching a diagnosis and improving the prognosis of those with a rare condition [3], concerns remain about the time taken to reach a diagnosis [7, 8], the level of care that may be available to individual patients and their carers [9–11] and the participation of patients in research [12]. These uncertainties prevent an optimal understanding of the illness, and can impede advanced care planning including palliative care [13]. The quality of life of the carers as well as the patients with a rare condition can be markedly affected [3, 14–16] but it is unclear as to the level of support that is available for the routine family. It is possible that studies that consult expert patient or professional groups are prone to some selection bias and some studies in the past may have been based on historical experience that predates the recent advances in diagnostics and therapeutics. Many studies have also had limited value due to their condition-specificity and not including a broader variety of rare conditions [14]. To understand the current impact of the rare condition on patients and families with rare conditions and the care they receive locally, there is a need to explore new methods that can continuously monitor patient/parented reported experience of the available support and clinical care as well as participation in research. In January 2017 the Office for Rare Conditions was founded in Glasgow, Scotland, following funding from the Glasgow Children’s Hospital Charity. The aim of the Office was to develop local solutions for raising awareness of rare conditions amongst healthcare professionals, to enhance support and clinical care of patients with rare conditions, and to promote participation in research. The aim of the current study was to measure these aspects at a specialist children’s hospital in the UK through a questionnaire survey. It is anticipated that the results would provide a current benchmark that can be used for improving local services for all people with rare conditions.

## Methods

In April 2018 the Office developed a questionnaire to determine the quality of care patients with rare conditions received at a tertiary children’s hospital in Glasgow. The questionnaire was developed following input from the Office’s Patient Advisory Group, the Steering Committee and external organisations including the Genetic Alliance and consisted of questions on six themes including: 1. diagnosis; 2. provision of information; 3. availability of support; 4. satisfaction with healthcare team; 5. awareness and support for life-limiting conditions; 6. participation in research (Table 1). The majority of questions had a binary response and could, therefore, be analysed quantitatively, however there was an option for comments following each question to enrich qualitative data. Further questions were added to the original questionnaire in January 2019 exploring theme 5; and in February 2020 questions were included on theme 6 (Table 1). The questionnaire did not collect any personally identifiable fields and had been locally approved as an evaluation of routine health care.

**Table 1:** Questionnaire. Contents of the questionnaire including the responses available and the date questions were added

Participant information	Responses available	Date added
Are you a patient or parent/carer of a child with a rare condition?	Patient	June 2018
	Parent/carer	
	Other—please specify	
Do you or your child attend a hospital in Glasgow? (Please specify)	Yes	June 2019
	No	
Theme 1: Diagnosis		
Have you or your child been formally diagnosed or is it suspected that you have a rare condition?	Formally diagnosed	June 2018
	Suspected to have a rare condition	
	Currently undiagnosed	
	If currently undiagnosed, how long has it been since you first reported your or your child's symptoms to a healthcare professional?	
Which rare condition do you or your child have or is it suspected that you or your child have? Please enter N/A if undiagnosed		June 2018
At what age did you or your child first develop symptoms?		June 2018
How long did it take to receive a diagnosis from the time you first approached a healthcare professional about your or your child's symptoms? Please enter N/A if undiagnosed		June 2018
Theme 2: Provision of Information		
At time of diagnosis, or suspected diagnosis, how much information were you given about the condition?	Lots of information was given	June 2018
	Adequate information was given	
	Little information was given	
	No information was given	
	N/A (currently undiagnosed)	
Where did/do you find information about this condition?	Through a healthcare professional	June 2018
	Through the literature/website of a patient support organisation	
	N/A (currently undiagnosed)	
	Other (please specify)	
Do you feel you have enough information on this condition?	Yes, I feel I know a lot about this condition	June 2018
	Yes, I have some information about this condition and am satisfied with what I know	
	No, I have access to some information, but would like to know more about this condition No, I don’t know anything about the condition Currently undiagnosed	
Theme 3: Availability of support		
Have you had the opportunity to meet another person/family with this condition (or someone who is in a similar situation, e.g. undiagnosed)?	Yes	June 2018
	No	
	If you haven’t, would you like to be given such an opportunity?	
How much support do you or your child receive in every day life from the following people? Family Hospital-based healthcare professional Your GP Other community-based healthcare professional School staff Friend	Lots	June 2018
	Some	
	Very occasional	
	Not at all	
	Not applicable	
Do you know of a support group/patient association for your condition or for undiagnosed conditions?	Yes	June 2018
	No	
Are you a member of this or any other patient support group?	Yes	June 2018
	No	
Do you feel well supported generally?	Yes	June 2018
	No	
Theme 4: Satisfaction with Healthcare Team		
With regards to you/your child's care, is there one specialist service that takes the lead?	Yes	June 2018
	No	
	If yes, which service at which hospital takes the lead	
How satisfied are you with the following? Having a consistent team of health professionals taking overall responsibility for you/your child’s health The overall support that you get from health professionals for you/your child Feeling that you are part of a health care team looking after you/your child How much health professionals know about you/your child’s condition Knowing which healthcare professional to contact for guidance/support with your/your child's condition	Extremely satisfied	June 2018
	Satisfied	
	Neither satisfied or dissatisfied	
	Dissatisfied	
	Extremely dissatisfied	
Theme 5: Awareness and support of limiting conditions		
Do you think your child has a life limiting condition	Yes	January 2019
	No	
	Don’t know	
If yes, have you received special support for that?	Yes	January 2019
	No	
	Don’t know	
If yes, what service has provided the support		January 2019
Theme 6: Participation in research		
Are you currently taking part in any research related to your condition?	Yes	February 2020
	No	
Would you be interested in taking part in any research regarding your condition?	Yes	February 2020
	No	

Between April 2018 and March 2020, the Office for Rare Conditions in Glasgow approached patients and carers who were in the outpatient waiting area, in the inpatient wards, who were visiting the Office’s exhibition stand in the Royal Hospital for Children, Glasgow or attending any education seminars organised by the Office and asked them to complete the above questionnaire in an electronic or a paper form. In addition, the electronic questionnaire was also promoted by the Office’s social media platforms including Facebook (@orcglasgow) and Twitter (@orcglasgow). Any patient with a rare condition, or parent/carer with a child diagnosed or awaiting diagnosis of a rare condition was eligible to complete the questionnaire if they were attending any hospital within the NHS Great Glasgow & Clyde Health Board. Although there were no exclusion criteria for completing the survey, the current report focuses on those patients who presented under the age of 18 years.

The quantitative aspects of the questionnaire were analysed using descriptive statistics. Qualitative responses were grouped and categorised.

## Results

Between June 2018 and March 2020, 130 questionnaires had been completed and returned on behalf of 134 people. Four parents answered the questionnaire in support of both of their affected children. Of the 130 questionnaires, 115 (88%) were completed by parents/carers, two (2%) were completed by grandparents, and the remaining 13 (10%) were completed by the patients themselves. Of the 130 questionnaires, six (5%) were not fully completed due to time constraints. No participants reported any difficulties with answering the questionnaire. Of the 56 who responded to the question about the location of their specialist care, 48 (86%) attended the Royal Hospital for Children in Glasgow.

### Diagnosis

Of the 134 patients, 114 (85%) had been formally diagnosed, 15 (11%) were undiagnosed and five (4%) had a suspected diagnosis. Of the 114 patients with a definitive diagnosis, all respondents disclosed their diagnosis and this included 103 different conditions, with nine patients who had more than one rare condition (Table 2). Of the 103 conditions, 27 (26%) of these were syndromal. Other organ systems included 16 musculoskeletal/dermatological (15%); 10 neurodevelopmental (10%); eight cardiovascular (8%); seven gastrointestinal (7%); seven neuromuscular (7%); six endocrine (6%); six oncological (6%); five haematological/ immune disorder (5%); four renal (4%); two lysosomal (2%); two metabolic (2%); one ENT (1%). one ophthalmological (1%); and one respiratory condition (1%).

**Table 2:** Description of the rare conditions that were encountered

Rare disease category	Specific rare disease	Diagnosis < 3 years old	Diagnosis between age 3–18 years old	Age of diagnosis unknown
Cardiovascular number of patients: 9	Aortic stenosis	X		
	Bicuspid aortic valve	X		
	CC1CNA1c heart defect	X		
	Coarctation of the aorta	X		
	Congenital heart disease	XX		
	Hypoplastic left heart	X		
	Unbalanced AVSD	X		
	WPW Syndrome	X		
Endocrine number of patients: 11	Addisons		X	X
	Hypercalcaemia	X		
	Hypospadias	X		
	MRKH			XXXXX
	PMDS	X		
	Pseudohypoparathyroidism	X	X	
ENT number of patients: 1	Deaf in right ear	X		
Gastrointestinal number of patients: 9	Achalasia		X	
	Alagilles	X		
	Chronic pseudo obstruction of large colon		X	
	Giant exomphalos	X		
	Hirschprungs disease	XXX		
	Congenital diaphragmatic hernia	X		
	Tracheo oesophageal fistula	X		
Haematological/immune disorder number of patients: 6	Antiphospholipid			X
	Autoimmune neutropenia		X	
	Chronic mucocutaneous candidiasis			X
	`HSP		X	
	Hypergammaglobulinaemia			X
Lysosomal number of patients: 4	Batten disease CLN2		X	X
	MPS 1—Hurler syndrome	XX		
Metabolic number of patients: 2	Glycogen storage disease IX	X		
	MCADD	X		
Musculoskeletal/dermatological number of patients: 22	Achondroplasia	X		
	Albinism		X	
	Arthrogyroposis	X		
	Crouzon	XXX		
	Epidermolysis bullosa		X	
	Erythromelalgia			XX
	Fibular hemimelia	X		
	Malignant infantile osteoporosis	X		
	Mandibulofacial dystosis with microcephaly		X	
	Marfan	X		
	Mixed connective tissue disorder		X	XX
	Popliteal pterygium syndrome	X		
	Raynauds			X
	Sjogrens			XX
	Talipes	X		
	Weaver syndrome		X	
Neurodevelopmental number of patients: 13	Brain AVM		X	
	Brain injury	X		
	Cornella de lange	X	XX	
	CTNNB1 syndrome		X	
	Joubert syndrome	X		
	PAK1 neurodevelopmental disorder		X	
	Periventricular leukomalacia	X		
	Rett syndrome	X	X	
	Riboflavin transporter deficiency		X	
	William syndrome	X		
Neuromuscular number of patients: 7	Adems disease		X	
	DMD		X	
	M.E.R.R.F			X
	Small fibre neuropathy			X
	Spinal muscle atrophy type 1	X		
	Sturge Weber	X		
	Worster drought syndrome	X		
Oncological number of patients: 8	ALL	X		
	Brain tumour		XX	
	Ewing sarcoma		XX	
	Medulloblastoma		X	
	Polycystic astrocytoma	X		
	Retinoblastoma		X	
Ophthalmological number of patients: 1	Bilateral congenital cataracts	X		
Renal number of patients: 4	Bartter syndrome	X		
	CKD	X		
	Nephrogenic DI	X		
	Nephrotic syndrome		X	
Respiratory number of patients: 1	NEH1 interstitial lung disease		X	
Syndrome number of patients: 37	Bardet Biedi syndrome		X	
	Beckwith-wiedemann syndrome	X		
	Chromosome 2 deletion	X		
	Chromosome 7 partial deletion			X
	Chromosome 8 disorder	X		
	Chromosome 8 disorder—short arm deletion, long arm duplication	X		
	CLTC chromosome abnormality		X	
	Deletion of 10p gene 13-15p	X		
	Di George	XX		
	Edwards/trisomy 18	X		
	Inverted duplication and deletion of 8p	X		
	Mosaic ring 14	X		
	Mowat Wilson syndrome	X		
	Noonan syndrome	X	X	
	Patau/trisomy 13	XX	X	
	Prader Willi			
	Primary ciliary dyskinesia	X	X	
	Treacher Collins syndrome	XX		
	Trichothiodystrophy	X		
	Trisomy 14	XXX		
	Tuberose sclerosis	XX	X	
	`Turner syndrome	X		
	Wolf-Hisrchhorn syndrome-9p		X	
	1Q43 deletion	X		
	16p11.2 micro deletion		X	
	22q11.2 deletion syndrome			X

The table below shows the 103 different rare conditions in the 114 patients with a formal diagnosis. Nine patients had more than one rare condition

*ALL* acute lymphoblastic leukaemia, *AVM* arteriovenous malformation, *AVSD* atrioventricular septal defect, *CKD* chronic kidney disease, *CTLC* cutaneous T-cell lymphoma, *DI* diabetes insipidus, *DMD* duchenne muscular dystrophy, *HSP* Henoch Schönlein purpura, *MCADD* medium chain acyl CoA dehydrogenase deficiency; *MPS 1* mucopolysaccharidosis type 1, *MRKH* Mayer Rokitansky Kuster Hauser Syndrome, *PMDS* Persistent Mullerian Duct Syndrome, *WPW* Wolff–Parkinson–White

### Age at first concern

**Fig. 1:**
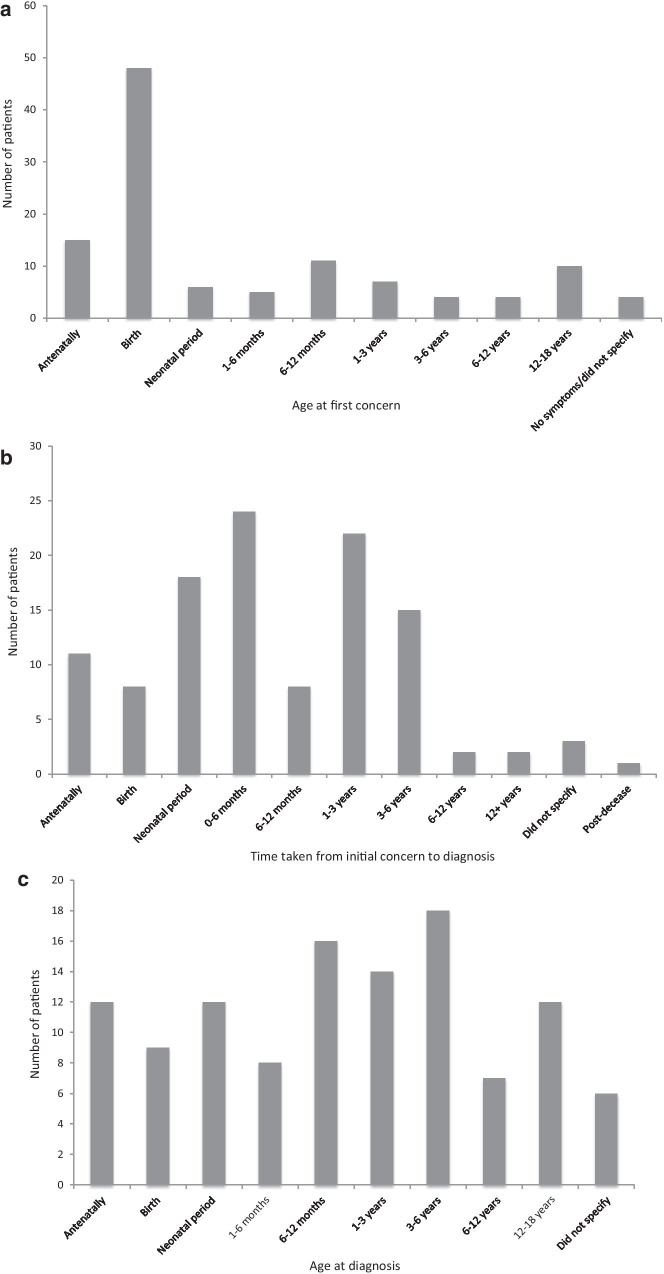
**a** Age at first concern. Participants who had a formal diagnosis were asked to report at which age they first developed concerns, n = 114. **b** Length of time taken to reach diagnosis from initial concern. Participants were asked to report the length of time taken from initial concern to obtaining a formal diagnosis, n = 114. **c** Age at formal diagnosis. The age at formal diagnosis was calculated following the responses to age of first concern and time taken to reach a diagnosis, n = 114

Of the 114 patients with a formal diagnosis, age at first concern ranged from the antenatal period to age 16 years of life (Fig. 1a). Symptoms/signs were first recognised at birth in 48 (42%) and there were antenatal concerns in 15 (13%). Of these 114, 4 (3.5%) had either no symptoms or did not specify the age at first concern.

### Time to diagnosis

The time taken to reach a formal diagnosis from initial concern ranged from antenatally to 17 years. Of the 114 patients 24 (21%) received a diagnosis within 0–6 months of developing symptoms, 22 (19%) obtained a diagnosis within 1–3 years, and 18 (16%) obtained a diagnosis in the neonatal period (Fig. 1b). A further three (3%) did not specify how long it took to obtain a diagnosis, and one patient (1%) was diagnosed following their death. Of those awaiting a formal diagnosis or who had a suspected diagnosis, five respondents did not specify the time they had been waiting for a diagnosis. In the remaining 15 the median time awaiting a formal diagnosis was 7 years (range, 0.5, 30).

### Age at diagnosis

Of the 114 patients with a formal diagnosis, 71 (62%) were diagnosed before the age of 3 years (Table 2). Of these 71 16 (23%) were diagnosed between the age of 6–12 months, 14 (20%) were diagnosed between the age of 1 and 3 years, and 12 (17%) were diagnosed both antenatally and neonatally (Fig. 1c). There were 37 patients who were diagnosed between the age of 3 and 18 years (Table 2). Of these 37, 18 (49%) were diagnosed between the age of 3 and 6 years, 7 (19%) were diagnosed between age of 6 and 12 years, and 12 (32%) were diagnosed between the age of 12–18 years. Six did not disclose their age at formal diagnosis.

### Provision of information

**Fig. 2:**
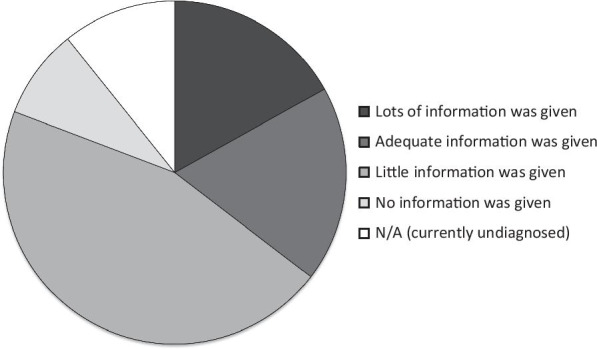
Provision of information at time of diagnosis. Participants stated how much information they were provided at the time of diagnosis, n = 130. The majority of patients were given no information (n = 59, 45%); 24 were given adequate information (18%); 22 were given lots of information (17%); and 11 were given no information at the time of diagnosis (8%)

At the time of diagnosis 59 (45%) of the 130 respondents felt they were provided little information about the condition, with 11 (8%) reporting they were given no information at all (Fig. 2). Information was received primarily from healthcare professionals in 45 (35%) respondents, this included the medical practitioners and the wider healthcare team, 29 (22%) received it from literature at patient support websites, and 15 (12%) received information from a mixture of both healthcare professionals and the literature at patient support websites. The remaining 29 (22%) with a formal diagnosis received the information from a range of sources including peer support groups, social media and other web-based resources. Two respondents (1.5%) had difficulty finding information, and one (0.8%) respondent could not find any information at all about their condition due to its rarity. Of the 130 respondents, 52 (40%) reported to be content with the information they had received whilst only 4 (3%) reported that they had limited knowledge on their condition.

### Availability of support

**Fig. 3:**
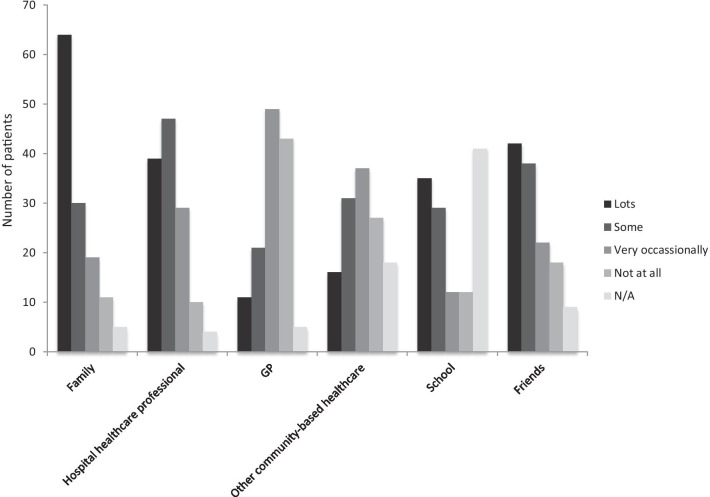
Availability of support. Participants were asked to rate availability of support from a wide variety of sources, n = 129

Of the 129 respondents to this section, 81 (63%) reported that they were well supported. This support was obtained from a range of sources to a variable extent. The majority of support was reported to have been received from family members, followed by friends, hospital services, school staff, other community-based health care services and lastly, primary care (Fig. 3). Of these, 43 (33%) reported that they did not receive any support from their primary health care practitioner and five (4%) did not feel seeking support from primary care was appropriate. Of the 129 respondents, 75 (58%) had met an individual or a family with the same condition and of the remaining 54, 18 (33%) would have liked the opportunity to meet another affected person or family, four people (7%) did not wish to, and 32 (59%) participants did not specify their preference. There were 81 (63%) respondents who were aware of a support group and 76 (59%) were also a member of a support group. Thus, over 90% of respondents who were aware of a support group were also a member of that support group.

### Satisfaction with the health care team

**Fig. 4:**
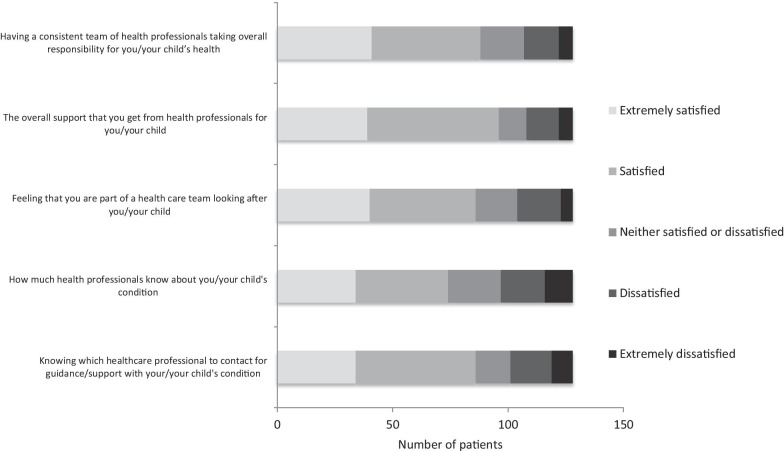
Satisfaction with healthcare team. Participants were asked to rate satisfaction with healthcare team in relation to different aspects of care, n = 128

Of the 127 respondents to this section, 78 (61%) could identify one specialist service that clearly took the lead for their or their child’s care and 86 (68%) knew of a named person who they could contact for their rare condition (Fig. 4). Of 128 respondents to the question on satisfaction with the health care team, 88 (69%) were either satisfied or extremely satisfied with the health care professionals taking overall responsibility for the clinical care of the rare condition and 96 (75%) reported to be well supported; 86 (67%) felt part of the team and 74 (58%) were satisfied with the knowledge that the health care team had about the rare condition.

### Awareness and support of life-limiting conditions

Of the 91 respondents to this section, 23 (25%) reported that they were aware that their child had a life-limiting condition and 30 (33%) did not know whether their child had a life-limiting condition. The remaining 38 (42%) did not feel their child had a life-limiting condition. Of the 23 respondents who felt their child had a life-limiting condition, only four (17%) reported to have received specialist support for this. Two (9%) had received specialist palliative support; one (4%) had access to clinical psychology, and the fourth had community support. A further two (9%) participants did not know whether they had received any support and the remaining 17 (74%) did not receive any support.

### Participation in research

Of the 17 respondents to this section, only four (24%) were actively participating in research whilst the remaining 13 (76%) all reported that they would be interested in taking part in future research.

## Discussion

The results of this survey provide a unique and objective insight into the health care experience of patients and their families at a regional specialist centre in the UK. It explores key topics including diagnostic timeframes, provision of information and support, wider healthcare team involvement, research participation and awareness and support for life-limiting conditions.

The results show that about 15% of the respondents were diagnosed within the neonatal period whilst a fifth had obtained a diagnosis within 6 months of developing symptoms and another fifth obtained a diagnosis within 1–3 years. With previous studies showing around 40% of patients having difficulty obtaining a diagnosis [14, 17, 18], the current study shows that 85% of the respondents received a diagnosis in childhood, and 80% of these diagnoses were within a shorter period than the 5-year time frame that is often reported [6, 19, 20]. Many previous studies have focussed only on a specific cohort of rare conditions and have, thereby, highlighted specific challenges associated with individual conditions. A strength of the current survey was the large range of rare conditions that had been covered, thus the results can be applied more generally. It is possible that several disease specific factors may have influenced the care outcomes examined in this study but an investigation of these factors was not within the scope of this work. There was no sample bias with only a maximum of five patients with the same condition. However, there may have been a selection bias as participants were recruited through social media platforms and via independent representatives approaching families for written responses.

Although the majority of participants did obtain a formal diagnosis within childhood, 4% had a suspected diagnosis and 11% were currently undiagnosed within the study. A delay in diagnosis can lead to patient or family stress and frustration, unnecessary investigations and disease progression [7, 8]. Factors that contribute to diagnostic delays include limited awareness amongst healthcare professionals and the public, inadequate testing opportunities, long waiting times and delays in obtaining results [8].

Patients with a rare condition should have a high standard of medical knowledge, easily accessible care pathways, research and treatment opportunities [2], yet this survey, along with some others [8, 21, 22] has shown the lack of information that parents had at initial diagnosis. Limited knowledge about their own condition can create major challenges for patients’ abilities to seek health care [23]. The current study confirmed this with over half of respondents who felt they were provided little or no information about the condition at the time of diagnosis, and only 40% reported to be content with the information they had received. Given that the majority of the affected children presented in early infancy, efforts for provision of information from the healthcare team at this critical period need to be intensified. This study considered the healthcare team as a whole, including the lead medical clinician and wider team, however looking at individual clinicians in their role in providing support and information at the time of diagnosis requires further study.

Patient support groups perform an increasingly vital role in the diagnosis and management of rare conditions, and are an important source of data surrounding these conditions [10, 24, 25]. Often they provide the sole source of information for patients and families [24]. It was interesting to note that over half of the respondents were a member of a support group and almost a quarter relied on peer support groups and social media rather than health care professionals as their primary source of information emphasising the critical role played by these groups, Other reports in similar settings suggest that membership of support groups may be less frequent [14]. Online patient networks can be a promising resource for peer support [26] and with increasing acceptability of technology across all ages [27] internet-based support groups may be of benefit for patients as well as their carers [28]. Given the wide range of conditions covered in this survey it is not surprising that a substantial proportion of respondents were not members of a support group and whether online support groups need to be particularly condition specific needs further exploration in the future.

Presently, there are very limited studies that explore the impact of rare conditions in children and their families [14]. The current survey explored the support from a variety of sources including family, the healthcare team, primary health care practitioners, other community based healthcare, schools and friends and showed that almost two-thirds of people felt well supported and highlighted the important role played by family members. Although the primary health care practitioner commonly serves as the point of access to specialist services in the UK, becoming familiar with all rare conditions is generally felt to be difficult especially in the primary care setting [9] and this can be compounded by the fact that often patients with rare conditions do not feel “unwell”, but perhaps just different [9] and care often involves multiple disciplines [15]. It was reassuring to note that a large majority of participants felt they had a consistent team of healthcare professionals taking overall responsibility of their condition and were satisfied with how much healthcare professionals know about their condition. It has often been suggested that the care of the patient with a rare condition is poorly coordinated [17]. It was, therefore, encouraging to discover that over two thirds of respondents felt part of the healthcare team in this survey and knew who to contact for guidance and the majority felt one specialist service had taken the lead. Overall, three quarters of respondents were satisfied with the support they received from healthcare professionals.

Support is essential at the time of diagnosis, however it is also crucial throughout the journey of a patient with a rare condition, particularly as many conditions can be life limiting or debilitating [5]. The current survey shows that 75% of families may not have a clear understanding of whether their child has a life limiting condition or not, perhaps reflecting on the lack of discussion on this topic as well as the gaps and barriers that may currently exist for the provision of palliative and end-of life care. Communication is a fundamental component of the palliative and end-of-life patient experience, with the term ‘palliative’ frequently misunderstood. It should be greater advocated that palliative care can also be given alongside traditional treatments [13], and the gap should be bridged between medical and palliative services, to allow for advanced care planning. Additionally, of those who felt their child did have a life-limiting condition, only a small percentage (17%) felt that they were being provided with specialist support for this.

Research is considered to be a vital aspect of improving the care of people with rare conditions [5, 14]. As the question enquiring whether patients/families’ involvement in research was added more recently in the survey, the response numbers were small, and only a quarter of respondents were actively taking part in research whilst the remainder were all keen on participating in research. There are many factors that could play a role in the limited participation, for example, lack of awareness of current research trials [17], selective recruitment criteria, geographical challenges, socioeconomic status [6, 12] or the time commitments when already caring for a child who may need around the clock care [5]. The present questionnaire could be adapted to explore this further and could even be used as a means of recruiting patients for future study. A recent study from 63 countries showed that just over one third of respondents actively participated in research (12) demonstrating that there is a global need for increased research participation.

## Conclusion

Although their diagnoses may differ, there are many commonalities experienced amongst families with rare conditions. Whilst the survey has shown that although there are several aspects of care where the results were more positive than expected, there are several indicators of care that require improvement. The indicators that we describe here can be considered to be key performance indicators that focus on the quality of care and may be of interest to reference centres within regional, national or international clinical networks such as European Reference Networks. The unique insight provided by this survey can be used as a platform to improve service delivery at a local level and can also act as a benchmark against which the quality of care can be compared across multiple centres.

## Data Availability

All raw data are available from the Office for Rare Condition subject to a Data Sharing Agreement.

## Abbreviations

ALL: Acute lymphoblastic leukaemia
AVM: Arteriovenous malformation
AVSD: Atrioventricular septal defect
CKD: Chronic kidney disease
CTLC: Cutaneous T-cell lymphoma
DI: Diabetes insipidus
DMD: Duchenne muscular dystrophy
HSP: Henoch Schönlein purpura
MCADD: Medium Chain Acyl CoA Dehydrogenase Deficiency
MPS 1: Mucopolysaccharidosis type 1
MRKH: Mayer Rokitansky Kuster Hauser Syndrome
PMDS: Persistent Mullerian Duct Syndrome
UK: United Kingdom
WPW: Wolff–Parkinson–White
